# Clinical benefit of graduated compression stockings for prevention of venous thromboembolism after total knee arthroplasty: post hoc analysis of a phase 3 clinical study of edoxaban

**DOI:** 10.1186/s12959-016-0087-z

**Published:** 2016-06-08

**Authors:** Takeshi Fuji, Satoru Fujita, Tetsuya Kimura, Kei Ibusuki, Kenji Abe, Shintaro Tachibana, Mashio Nakamura

**Affiliations:** Department of Orthopedic Surgery, Japan Community Healthcare Organization Osaka Hospital, 4-2-78 Fukushima, Fukushima-ku, Osaka 553-0003 Japan; Department of Orthopedic Surgery, Takarazuka Daiichi Hospital, 19-5 Kogetsu-cho, Takarazuka, 665-0832 Japan; Daiichi Sankyo Co., Ltd., 3-5-1, Nihonbashi Honcho, Chuo-ku, Tokyo, 103-8426 Japan; Clinical Data and Biostatistics Department, Daiichi Sankyo Co., Ltd., 1-2-58, Hiromachi, Shinagawa-ku, Tokyo, 140-8710 Japan; Department of Orthopedic Surgery, Mishuku Hospital, 5-33-12 Shimomeguro, Meguro-ku, Tokyo, 153-0051 Japan; Department of Cardiology and Nephrology, Mie University Graduate School of Medicine, 2-174 Edobashi, Tsu, Tsu, 514-8507 Mie Japan

**Keywords:** Venous thromboembolism, Deep vein thrombosis, Intermittent pneumatic compression, Graduated compression stockings, Edoxaban, Enoxaparin, Total knee arthroplasty, Orthopedic surgery, VTE, DVT

## Abstract

**Background:**

Guidelines from the Japanese Circulation Society recommend prophylaxis with anticoagulation plus intermittent pneumatic compression or graduated compression stockings (GCS) among patients at the highest risk for developing venous thromboembolism (VTE). However, the benefits of concomitant GCS use for patients undergoing total knee arthroplasty (TKA) and receiving anticoagulation remain unknown. In this study, the efficacy of GCS plus anticoagulation compared with anticoagulation alone was evaluated among patients undergoing TKA.

**Methods:**

This study is a post hoc analysis of a previously reported phase 3 trial involving patients undergoing TKA. In the primary study, which permitted the use of GCS for mechanical prophylaxis, patients were randomized to receive edoxaban 30 mg once daily or enoxaparin 20 mg twice daily for 11 to 14 days following TKA. The primary endpoint was the incidence of VTE, a composite of symptomatic deep vein thrombosis (DVT), symptomatic pulmonary embolism (PE), and asymptomatic DVT. Treatment comparisons were performed using the chi-square test, and the 95 % confidence intervals were calculated.

**Results:**

Among patients receiving edoxaban, the incidence of VTE was 3.8 and 5.8 % for patients with and without GCS, respectively. For patients receiving enoxaparin, VTE incidence was 8.4 and 20.8 % among those with and without GCS, respectively. Overall, VTE incidence was 6.0 and 13.0 % for anticoagulated patients with and without GCS mechanical prophylaxis, respectively. No deaths or symptomatic PE were reported during this study.

**Conclusions:**

Although the incidence of VTE was >2-fold lower among patients receiving anticoagulation plus GCS compared with those receiving anticoagulation alone, statistical significance was not achieved. Further studies are required to confirm the findings of this preliminary analysis.

**Trial registration:**

ClinicalTrials.gov Identifier: NCT01181102

## Background

Major lower limb orthopedic surgery—such as total knee arthroplasty (TKA), total hip arthroplasty (THA), and hip fracture surgery (HFS)—is a significant risk factor for postoperative venous thromboembolism (VTE), encompassing deep vein thrombosis (DVT) and pulmonary embolism (PE) [1, 2]. For patients undergoing lower limb orthopedic surgery, several pharmacological and mechanical therapies are available for the prevention of VTE, including graduated compression stockings (GCS), which are a convenient and inexpensive mechanical prophylaxis measure [1]. Although the exact mechanism of action of GCS remains unclear, they are believed to reduce the total cross-sectional area of the leg veins, thereby increasing venous blood flow velocity and preventing venous stasis in the lower limbs [3].

Guidelines from the Japanese Circulation Society recommend the use of GCS for prophylaxis among patients at an intermediate risk for VTE [1]. Anticoagulation or intermittent pneumatic compression (IPC) is recommended for patients at a high risk for VTE, such as those undergoing major orthopedic surgery, while anticoagulation plus IPC or GCS is recommended for patients at the highest risk for VTE [1]. However, the efficacy of the combined treatment of anticoagulation with GCS relative to anticoagulation alone for VTE prevention among patients undergoing major lower limb orthopedic surgery has not been evaluated. In this study, an exploratory analysis was performed to evaluate the efficacy of GCS combined with enoxaparin or edoxaban, a direct, oral, factor Xa inhibitor, using data from a phase 3 clinical study that assessed the efficacy and safety of edoxaban (vs enoxaparin) for the prevention of postoperative VTE in patients undergoing TKA.

## Methods

### Study selection

Three phase 3 studies involving patients undergoing TKA (TKA P3; STARS E-3 trial) [4], THA (THA P3; STARS J-5 trial) [5], or HFS (HFS P3; STARS J-4 trial) [6] were considered for inclusion in this analysis. A total of 716, 610, and 92 patients were enrolled in the TKA P3 [4], THA P3 [5], and HFS P3 [6] studies, respectively, of whom 594, 503, and 73, respectively, were evaluable for efficacy. These 3 studies were conducted between October 2008 and January 2010. In all 3 studies, the concomitant use of any method of mechanical prophylaxis, including GCS and IPC, was permitted. Table 1 summarizes the number of patients available for efficacy analysis stratified by the method of mechanical prophylaxis. Since there were few patients without mechanical prophylaxis in the THA P3 (1 patient) and HFS P3 (9 patients) studies, these 2 studies were excluded from the present analysis. In TKA P3, 129 patients received GCS alone as mechanical prophylaxis and 104 patients had no mechanical prophylaxis. Data from the TKA P3 study were included in the present analysis.

**Table 1:** Use of mechanical prophylaxis (efficacy analysis set)

Mechanical prophylaxis		TKA P3 *N* = 594	THA P3 *N* = 503	HFS P3 *N* = 73
GCS	IPC			
o	x	129	136	39
o	o	238	274	15
x	o	123	92	10
x	x	104	1	9

*GCS* graduated compression stockings, *HFS P3* hip fracture surgery phase 3 study, *IPC* intermittent pneumatic compression, *THA P3* total hip arthroplasty phase 3 study, *TKA P3* total knee arthroplasty phase 3 study

o = patients receiving indicated treatment; x = patients without the indicated treatment

### Study design and site selection

**Fig. 1:**
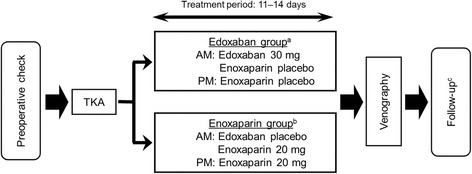
Study design (phase 3 total knee arthroplasty study) [4]. ^a^Treatment with oral edoxaban commenced 6–24 h postoperatively. ^b^Treatment with subcutaneous enoxaparin commenced 24–36 h postoperatively. ^c^Follow-up occurred 25–35 days after the last administered dose

TKA P3 was a randomized, double-blind, parallel-group study performed by the double-dummy method (Fig. 1) [4]. Edoxaban (30 mg) was administered orally once a day, while enoxaparin (20 mg) was injected subcutaneously twice a day for 11 to 14 days in accordance with standard practice. Edoxaban was administered 6 to 24 h after surgery, whereas enoxaparin treatment commenced 24 to 36 h following surgery. Venography was performed within 24 h after the last dose, and occurrence of VTE was determined by an independent Clinical Event Committee that was blinded to the treatment of the patients. The primary efficacy endpoint was the percentage of patients who developed asymptomatic DVT, symptomatic DVT, or symptomatic PE (the incidence of VTE).

In TKA P3, 716 patients were enrolled at 71 sites. To exclude possible bias due to selection of GCS depending on the severity of illness or the VTE risk of each patient, 18 sites where GCS had been used for selected patients were excluded from the present analysis. Thus, the remaining 422 patients from 53 sites were considered for inclusion in the present analysis. To avoid the influence of other mechanical methods (eg, IPC), patients who had also used IPC were excluded as well. Thus, the present analysis compared 101 patients who had used GCS alone as mechanical prophylaxis (53 from the edoxaban group and 48 from the enoxaparin group) with 100 patients without any mechanical prophylaxis (52 from the edoxaban group and 48 from the enoxaparin group), so a total of 201 patients were included in this assessment of the efficacy of GCS.

### Statistical analysis

The incidence of VTE was summarized using descriptive statistics. Treatment comparisons were made using the *χ*2 test and the 95 % confidence interval was calculated.

## Results

The demographics and baseline characteristics of patients included in this subanalysis are presented in Table 2. The mean age, body weight, and creatinine clearance were not different among patients regardless of anticoagulant and/or mechanical prophylaxis use. Tourniquet use was lower, whereas the use of epidural and lumbar anesthesia was higher among patients receiving GCS relative to those without any mechanical prophylaxis. This difference was due to facility-specific practices; facilities employing GCS for prophylaxis had lower rates of tourniquet or general anesthesia use. Mean GCS use was 9.5 and 9.6 days for the edoxaban and enoxaparin groups, respectively. The mean (standard deviation) time interval between surgery and the start of postoperative anticoagulant therapy was 20 h and 7 min (2 h and 50 min) for the edoxaban group and 28 h and 54 min (3 h and 29 min) for the enoxaparin group. Enoxaparin was administered between 24 to 36 h after surgery, in accordance with enoxaparin prescribing practices [7], accounting for this difference in initiation of anticoagulation following surgery.

**Table 2:** Demographic and baseline characteristics summary

Demographic and other baseline characteristics	No mechanical prophylaxis		GCS alone	
	*N* = 100		*N* = 101	
	Edoxaban	Enoxaparin	Edoxaban	Enoxaparin
	*N* = 52	*N* = 48	*N* = 53	*N* = 48
Sex, n (%)				
Male	11 (21.2)	9 (18.8)	8 (15.1)	9 (18.8)
Female	41 (78.8)	39 (81.3)	45 (84.9)	39 (81.3)
Age, (y)				
Mean ± SD	70.9 ± 7.9	71.7 ± 7.8	73.5 ± 6.1	73.2 ± 7.0
Weight, (kg)				
Mean ± SD	63.2 ± 10.2	64.3 ± 11.9	56.8 ± 10.2	60.3 ± 10.1
CL_cr_, (mL/min)				
Mean ± SD	78.3 ± 24.6	76.2 ± 26.5	71.1 ± 20.1	74.9 ± 20.0
Duration of surgery, (h:min)				
Mean ± SD	2:03 ± 0:44	1:59 ± 0:35	1:42 ± 0:26	1:45 ± 0:24
Tourniquet use during surgery, n (%)	50 (96.2)	46 (95.8)	42 (79.2)	38 (79.2)
Method of anesthesia, n (%)				
General anesthesia	43 (82.7)	35 (72.9)	24 (45.3)	20 (41.7)
Epidural anesthesia	8 (15.4)	7 (14.6)	17 (32.1)	14 (29.2)
Lumbar anesthesia	24 (46.2)	24 (50.0)	38 (71.7)	34 (70.8)
Duration from the end of surgery to the start of anticoagulation, (h:min)				
Mean ± SD	20:31 ± 2:39	30:41 ± 3:26	19:43 ± 2:59	27:08 ± 2:30
Duration from surgery to ambulation (days)				
Mean ± SD	2.2 ± 0.7	2.4 ± 0.8	3.6 ± 1.4	3.8 ± 2.1
Duration of treatment (days)				
Mean ± SD	12.1 ± 1.6	11.6 ± 2.6	12.4 ± 1.6	12.4 ± 1.7
Duration of GCS use (days)				
Mean ± SD	-	-	9.5 ± 2.7	9.6 ± 2.7

*CL*_*cr*_ creatinine clearance, *GCS* graduated compression stockings, *SD* standard deviation

In the edoxaban group, the incidence of VTE was 3.8 % (2/53) and 5.8 % (3/52) for patients with and without GCS mechanical prophylaxis, respectively (*p* = 0.6312; Table 3). In the enoxaparin group, the incidence of VTE was 8.4 % (4/48) for patients using GCS alone as mechanical prophylaxis, and 20.8 % (10/48) for patients without mechanical prophylaxis (*p* = 0.0827). Combining the 2 groups, the incidence of VTE was 6.0 % (6/101) and 13.0 % (13/100) for patients with and without GCS mechanical prophylaxis. There were no deaths in this study. Of the 201 patients included in this analysis, no instances of symptomatic or asymptomatic PE and no instances of symptomatic DVT were observed; all patients had asymptomatic DVT.

**Table 3:** Incidence of VTE^a^ in patients using GCS for prophylaxis

	Edoxaban	Enoxaparin	Total
	*N* = 105	*N* = 96	*N* = 201
No mechanical prophylaxis	5.8 % (3/52)	20.8 % (10/48)	13.0 % (13/100)
Mechanical prophylaxis with GCS alone	3.8 % (2/53)	8.4 % (4/48)	6.0 % (6/101)
Chi-square test	*p* = 0.6312	*p* = 0.0827	*p* = 0.0872
Difference between with and without GCS treatment groups	–2.0	–12.5	–7.1
95 % CI of the difference shown by the score test	−12.2, 7.8	−26.8, 1.9	−15.7, 1.2

*CI* confidence interval, *GCS* graduated compression stockings, *VTE* venous thromboembolism

^a^All instances of VTE were asymptomatic DVT; no instances of symptomatic or asymptomatic PE or symptomatic DVT were observed

## Discussion

Anticoagulants prevent VTE by suppressing hypercoagulability [8], while GCS prevent VTE by increasing the venous blood flow velocity and impeding venous stasis in the legs [3]. Thus, 2 independent elements of Virchow’s thrombogenic triad (“hypercoagulability” and “venous stasis”) [8] are targeted when prophylaxis with anticoagulant therapy and GCS is combined. This is thought to be the mechanism by which combined therapy achieves a greater reduction in the incidence of VTE after major lower limb orthopedic surgery. Few studies have examined the efficacy of combined anticoagulant and GCS therapy relative to anticoagulation alone for VTE prevention among patients undergoing major orthopedic surgery [9]. A study involving patients undergoing THA in the UK revealed that the incidence of DVT or PE was not significantly different between patients receiving enoxaparin plus GCS compared to those receiving enoxaparin alone [10]. However, this study was limited by its small sample size, with 32 patients randomized to each treatment group [10]. Among Swedish patients undergoing THA, combined treatment with dextran (infused at anesthesia and for 2 days following surgery) and GCS was more effective for postoperative DVT prevention than treatment with dextran alone [11].

Although Japanese guidelines recommend the use of GCS with anticoagulation for prophylaxis among patients at the highest risk for VTE [1], the efficacy of this combined treatment relative to anticoagulation alone for Japanese patients undergoing major orthopedic surgery has previously not been reported. The findings from the present study suggest that concomitant treatment with anticoagulation and GCS resulted in a >2-fold reduction in VTE incidence relative to anticoagulation alone among Japanese patients undergoing TKA, although this difference was not statistically significant. Addition of GCS therapy led to a numerically greater reduction in VTE incidence primarily among patients receiving enoxaparin, while this reduction was less pronounced among those receiving edoxaban. This difference may have arisen partly because edoxaban was started earlier in the postoperative period; as a result, there was a smaller margin for further reduction of VTE occurrence with the addition of GCS among patients treated with edoxaban. Given the limited number of patients in this analysis (*n* = 201), and the delay in the initiation of anticoagulation with enoxaparin relative to edoxaban, these exploratory results need to be confirmed in larger, prospective, randomized trials.

## Conclusion

To evaluate the efficacy of GCS for prevention of VTE, an exploratory analysis was performed by using data from a phase 3 clinical study of postoperative prophylaxis with edoxaban vs enoxaparin in patients undergoing TKA. The results of the analysis suggested that the occurrence of VTE was numerically lower among patients receiving anticoagulant plus GCS therapy relative to those receiving anticoagulation alone, although this difference was not statistically significant. Further studies are required to confirm the findings of this preliminary analysis. Additionally, due to the limitations of this analysis, further studies need to be performed to evaluate the relative prophylactic efficacy of combined anticoagulant and GCS therapy compared with anticoagulation alone.

## References

[1] JCS Joint Working Group (2011). Guidelines for the diagnosis, treatment and prevention of pulmonary thromboembolism and deep vein thrombosis (JCS 2009). Circ J.

[2] Anderson FA, Spencer FA (2003). Risk factors for venous thromboembolism. Circulation.

[3] Laryea J, Champagne B (2013). Venous thromboembolism prophylaxis. Clin Colon Rectal Surg.

[4] Fuji T, Wang CJ, Fujita S, Kawai Y, Nakamura M, Kimura T (2014). Safety and efficacy of edoxaban, an oral factor Xa inhibitor, versus enoxaparin for thromboprophylaxis after total knee arthroplasty: the STARS E-3 trial. Thromb Res.

[5] Fuji T, Fujita S, Kawai Y, Nakamura M, Kimura T, Fukuzawa M (2015). Efficacy and safety of edoxaban versus enoxaparin for the prevention of venous thromboembolism following total hip arthroplasty: STARS J-V. Thromb J.

[6] Fuji T, Fujita S, Kawai Y, Nakamura M, Kimura T, Kiuchi Y (2014). Safety and efficacy of edoxaban in patients undergoing hip fracture surgery. Thromb Res.

[7] Clexane® for Subcutaneous Injection Kit 2000 IU. Tokyo, Japan: Sanofi-Aventis K.K; 2012 [Package Insert (Ver. 8), in Japanese].

[8] Turpie AG, Esmon C (2011). Venous and arterial thrombosis-pathogenesis and the rationale for anticoagulation. Thromb Haemost.

[9] Sachdeva A, Dalton M, Amaragiri SV, Lees T (2010). Elastic compression stockings for prevention of deep vein thrombosis. Cochrane Database Syst Rev.

[10] Kalodiki EP, Hoppensteadt DA, Nicolaides AN, Fareed J, Gill K, Regan F (1996). Deep venous thrombosis prophylaxis with low molecular weight heparin and elastic compression in patients having total hip replacement. A randomised controlled trial. Int Angiol.

[11] Ohlund C, Fransson SG, Starck SA (1983). Calf compression for prevention of thromboembolism following hip surgery. Acta Orthop Scand.

